# 2,3-Dichloro­pyridine

**DOI:** 10.1107/S1600536811030261

**Published:** 2011-08-02

**Authors:** Li-Juan Luo, Jian-Quan Weng

**Affiliations:** a Department of Chemical Engineering, Ningbo University of Technology, Ningbo 315016, People’s Republic of China; b College of Chemical Engineering and Materials Science, Zhejiang University of Technology, Hangzhou 310014, People’s Republic of China

## Abstract

The complete mol­ecule of the title compound, C_5_H_3_Cl_2_N, is generated by crystallographic twofold symmetry, which forces the pyridine N atom and the opposite C—H group to be statistically disordered. In the crystal, weak aromatic π–π stacking [centroid–centroid separation = 3.805 (4) Å and slippage = 1.704 Å] leads to [100] stacks of mol­ecules. Short Cl⋯Cl contacts [3.334 (3) Å] are also observed.

## Related literature

For the biological activity of related compounds, see: Liu *et al.* (2011 ▶). For related structures, see: Ma *et al.* (2007 ▶), Liu & Liu (2011 ▶).
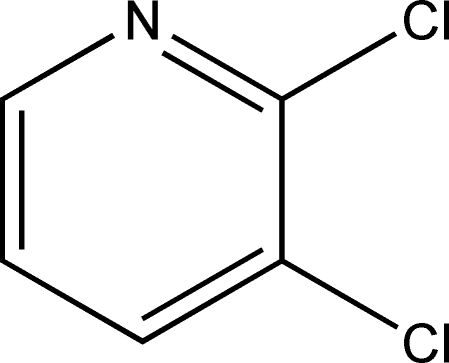


## Experimental

### 

#### Crystal data

C_5_H_3_Cl_2_N
*M* *_r_* = 147.98
Monoclinic, 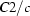

*a* = 3.805 (3) Å
*b* = 14.196 (12) Å
*c* = 10.68 (1) Å
β = 97.221 (14)°
*V* = 572.3 (9) Å^3^
*Z* = 4
Mo *K*α radiation
μ = 1.00 mm^−1^
*T* = 113 K
0.36 × 0.04 × 0.04 mm

#### Data collection

Rigaku Saturn CCD diffractometer
Absorption correction: multi-scan (*CrystalClear*; Rigaku/MSC, 2005 ▶) *T*_min_ = 0.714, *T*_max_ = 0.961
2936 measured reflections
675 independent reflections
541 reflections with *I* > 2σ(*I*)
*R*_int_ = 0.044

#### Refinement

*R*[*F* ^2^ > 2σ(*F* ^2^)] = 0.025
*wR*(*F* ^2^) = 0.054
*S* = 1.01
675 reflections
43 parameters
2 restraints
All H-atom parameters refined
Δρ_max_ = 0.31 e Å^−3^
Δρ_min_ = −0.19 e Å^−3^

### 

Data collection: *CrystalClear* (Rigaku/MSC, 2005 ▶); cell refinement: *CrystalClear*; data reduction: *CrystalClear*; program(s) used to solve structure: *SHELXS97* (Sheldrick, 2008 ▶); program(s) used to refine structure: *SHELXL97* (Sheldrick, 2008 ▶); molecular graphics: *SHELXTL* (Sheldrick, 2008 ▶); software used to prepare material for publication: *CrystalStructure* (Rigaku/MSC, 2005 ▶).

## Supplementary Material

Crystal structure: contains datablock(s) global, I. DOI: 10.1107/S1600536811030261/hb5953sup1.cif

Structure factors: contains datablock(s) I. DOI: 10.1107/S1600536811030261/hb5953Isup2.hkl

Supplementary material file. DOI: 10.1107/S1600536811030261/hb5953Isup3.cml

Additional supplementary materials:  crystallographic information; 3D view; checkCIF report

## Figures and Tables

**Fig. 1.**
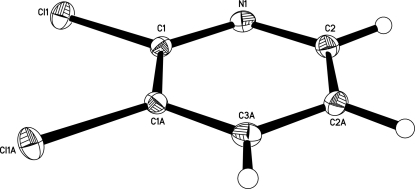
The molecular structure of (I). Displacement ellipsoids are drawn at the 30% probability level and H atoms are shown as small spheres of arbitrary radii. Atoms with suffix A are generated by (1–x, y, 3/2–z). Just one orientation of N1 and C3 is shown.

**Fig. 2.**
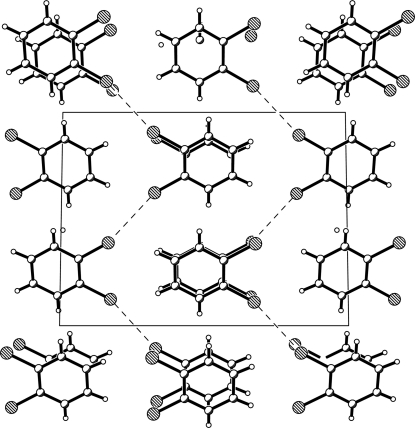
The crystal packing for (I) with short Cl···Cl contacts indicated by dashed lines.

## supplementary crystallographic information

### Comment

Pyridine derivatives are valuable intermidiates and various biological activities. the structure of 2,3-dichloropyridine was confirmed by X-ray crstallography. For biological activities of related compounds, see: Liu *et al.* (2011). For related structure, see: Ma *et al.* (2007), Liu *et al.* & Liu (2011);

Single-crystal X-ray diffraction analysis reveals that the title compound crystallizes in the monoclinic space group *C*2/*c*. As shown in Fig. 1, the pyridine ring is nearly planar [mean deviation = 0.003 Å]. As shown in Fig. 2, the crystal structure is stabilized by van der Waals' interactions.

### Experimental

2,3-dichloropyridine is commercially available. Colourless prisms were grown from ethanol.

### Refinement

All the H atoms were positioned geometrically (C—H = 0.93Å) and refined as riding with *U*_iso_(H) = 1.2*U*_eq_(C)

### Crystal data

**Table:**

C_5_H_3_Cl_2_N	*F*(000) = 296
*M**_r_* = 147.98	*D*_x_ = 1.717 Mg m^−^^3^
Monoclinic, *C*2/*c*	Mo *K*α radiation, λ = 0.71073 Å
Hall symbol: -C 2yc	Cell parameters from 985 reflections
*a* = 3.805 (3) Å	θ = 1.9–27.8°
*b* = 14.196 (12) Å	µ = 1.00 mm^−^^1^
*c* = 10.68 (1) Å	*T* = 113 K
β = 97.221 (14)°	Prism, colorless
*V* = 572.3 (9) Å^3^	0.36 × 0.04 × 0.04 mm
*Z* = 4	

### Data collection

**Table:**

Rigaku Saturn CCD diffractometer	675 independent reflections
Radiation source: rotating anode	541 reflections with *I* > 2σ(*I*)
multilayer	*R*_int_ = 0.044
Detector resolution: 14.63 pixels mm^-1^	θ_max_ = 27.8°, θ_min_ = 2.9°
ω and φ scans	*h* = −4→4
Absorption correction: multi-scan (*CrystalClear*; Rigaku/MSC, 2005)	*k* = −18→18
*T*_min_ = 0.714, *T*_max_ = 0.961	*l* = −13→13
2936 measured reflections	

### Refinement

**Table:**

Refinement on *F*^2^	Primary atom site location: structure-invariant direct methods
Least-squares matrix: full	Secondary atom site location: difference Fourier map
*R*[*F*^2^ > 2σ(*F*^2^)] = 0.025	Hydrogen site location: inferred from neighbouring sites
*wR*(*F*^2^) = 0.054	All H-atom parameters refined
*S* = 1.01	*w* = 1/[σ^2^(*F*_o_^2^) + (0.022*P*)^2^] where *P* = (*F*_o_^2^ + 2*F*_c_^2^)/3
675 reflections	(Δ/σ)_max_ = 0.001
43 parameters	Δρ_max_ = 0.31 e Å^−^^3^
2 restraints	Δρ_min_ = −0.19 e Å^−^^3^

### Special details

**Table:**

Geometry. All e.s.d.'s (except the e.s.d. in the dihedral angle between two l.s. planes) are estimated using the full covariance matrix. The cell e.s.d.'s are taken into account individually in the estimation of e.s.d.'s in distances, angles and torsion angles; correlations between e.s.d.'s in cell parameters are only used when they are defined by crystal symmetry. An approximate (isotropic) treatment of cell e.s.d.'s is used for estimating e.s.d.'s involving l.s. planes.
Refinement. Refinement of *F*^2^ against ALL reflections. The weighted *R*-factor *wR* and goodness of fit *S* are based on *F*^2^, conventional *R*-factors *R* are based on *F*, with *F* set to zero for negative *F*^2^. The threshold expression of *F*^2^ > σ(*F*^2^) is used only for calculating *R*-factors(gt) *etc*. and is not relevant to the choice of reflections for refinement. *R*-factors based on *F*^2^ are statistically about twice as large as those based on *F*, and *R*- factors based on ALL data will be even larger.

### Fractional atomic coordinates and isotropic or equivalent isotropic displacement parameters (Å^2^)

**Table:**

	*x*	*y*	*z*	*U*_iso_*/*U*_eq_	Occ. (<1)
Cl1	0.26918 (10)	0.32961 (3)	0.61537 (4)	0.02346 (14)	
C1	0.3983 (4)	0.43421 (10)	0.69098 (13)	0.0154 (3)	
C2	0.2951 (3)	0.51548 (10)	0.63275 (12)	0.0182 (3)	0.50
H2	0.138 (6)	0.514 (2)	0.5570 (16)	0.022*	0.50
N1	0.2951 (3)	0.51548 (10)	0.63275 (12)	0.0182 (3)	0.50
C3	0.3971 (4)	0.59762 (10)	0.69191 (15)	0.0214 (4)	
H3	0.312 (4)	0.6559 (8)	0.6561 (15)	0.026*	

### Atomic displacement parameters (Å^2^)

**Table:**

	*U*^11^	*U*^22^	*U*^33^	*U*^12^	*U*^13^	*U*^23^
Cl1	0.0279 (3)	0.0201 (2)	0.0216 (2)	−0.00436 (16)	0.00026 (17)	−0.00631 (16)
C1	0.0142 (8)	0.0163 (7)	0.0160 (7)	−0.0009 (6)	0.0031 (6)	−0.0027 (6)
C2	0.0158 (8)	0.0235 (7)	0.0149 (7)	−0.0009 (6)	0.0008 (6)	0.0016 (6)
N1	0.0158 (8)	0.0235 (7)	0.0149 (7)	−0.0009 (6)	0.0008 (6)	0.0016 (6)
C3	0.0184 (9)	0.0185 (8)	0.0269 (9)	0.0015 (6)	0.0012 (7)	0.0073 (7)

### Geometric parameters (Å, °)

**Table:**

Cl1—C1	1.7317 (18)	C2—H2	0.943 (10)
C1—C2	1.346 (2)	C3—C3^i^	1.382 (3)
C1—C1^i^	1.394 (3)	C3—H3	0.951 (9)
C2—C3	1.359 (2)		
			
C2—C1—C1^i^	120.97 (9)	C3—C2—H2	122.4 (18)
C2—C1—Cl1	118.07 (12)	C2—C3—C3^i^	120.92 (9)
C1^i^—C1—Cl1	120.96 (6)	C2—C3—H3	119.8 (10)
C1—C2—C3	118.10 (14)	C3^i^—C3—H3	119.1 (10)
C1—C2—H2	119.2 (18)		
			
C1^i^—C1—C2—C3	0.5 (3)	C1—C2—C3—C3^i^	0.5 (3)
Cl1—C1—C2—C3	−179.84 (12)		

Symmetry codes: (i) −*x*+1, *y*, −*z*+3/2.
